# Vitamin B12 and Affective Disorders: A Focus on the Gut-Brain Axis

**DOI:** 10.31083/AP49138

**Published:** 2025-12-16

**Authors:** Chenyue Xu, Lingzhuo Kong, Tingting Mou, Anying Tang, Shaohua Hu, Jianbo Lai

**Affiliations:** ^1^Department of Psychiatry, The First Affiliated Hospital, Zhejiang University School of Medicine, 310003 Hangzhou, Zhejiang, China; ^2^Nanhu Brain-Computer Interface Institute, 310003 Hangzhou, Zhejiang, China; ^3^The Zhejiang Key Laboratory of Precision Psychiatry, 310003 Hangzhou, Zhejiang, China; ^4^MOE Frontier Science Center for Brain Science and Brain-Machine Integration, Zhejiang University School of Medicine, 310003 Hangzhou, Zhejiang, China; ^5^Brain Research Institute of Zhejiang University, 310003 Hangzhou, Zhejiang, China; ^6^Department of Psychology and Behavioral Sciences, Zhejiang University, 310003 Hangzhou, Zhejiang, China; ^7^Zhejiang Engineering Center for Mathematical Mental Health, 310003 Hangzhou, Zhejiang, China

**Keywords:** vitamin B12, bipolar disorder, depressive disorder, gastrointestinal microbiome

## Abstract

Accumulating evidence highlights the role of Vitamin B12 (VitB12) in the pathophysiology of affective disorders. However, its influence on brain function and the underlying mechanisms remain incompletely understood. In humans, VitB12 is obtained solely from dietary sources, primarily animal-based foods. VitB12 deficiency leads to the accumulation of homocysteine, a known contributor to emotional and behavioral dysregulation. VitB12 plays a critical role in maintaining neuron stability, synapsis plasticity, and regulating neuroinflammation by modulating key bioactive factors. These processes help to alleviate hippocampal damage, mitigate blood-brain barrier disruption, reduce oxidative stress, and enhance both structural and functional connectivity—collectively contributing to resilience against affective disorders. VitB12 from both diet and microbial sources is essential to gut homeostasis. Within the gut lumen, it stabilizes gut microbial communities, promotes short-chain fatty acid (SCFA) production, and supports neurotransmitter metabolism (e.g., serotonin and dopamine) via its role in S-adenosyl-l-methionine biosynthesis. Crucially, VitB12, gut microbiota, SCFAs, intestinal mucosa, and vagal nerve signaling interact synergistically within the gut-brain axis (GBA) to maintain gut microenvironment stability, protect the gut-blood barrier, and suppress neuroinflammatory cascades, eventually reducing the susceptibility to affective disorders. This review synthesizes current evidence on the involvement of VitB12 in the GBA, its association with mood regulation, and its potential as a nutritional adjunct in managing affective disorders. By elucidating this integrative mechanism, we provide new insights into targeting the GBA to improve clinical outcomes in affective disorders.

## Main Points

• Draw a map of vitamin B12 in the gut-brain axis with the latest findings.

• Link vitamin B12 to affective disorders via systematic circulation and the gut-brain axis.

• Probe the potential of vitamin B12 to influence resilience to affective disorders.

## 1. Introduction

Affective disorders, mainly including major depressive disorder (MDD) and bipolar disorder (BD), remain significant contributors to the substantial global health burden due to their high prevalence, recurrence, and suboptimal clinical outcomes in the absence of timely interventions. According to global health data, MDD and BD ranked 13th and 67th, respectively, in disability-adjusted life years across all age groups. Strikingly, among individuals aged 15–24 years, they rose to 4th and 32nd, underscoring their disproportionate impact on youth population [1]. Despite extensive research efforts, the etiology and pathophysiological mechanisms underlying affective disorders remain incompletely understood, posing a major challenge for the development of targeted therapies.

**Fig. 1.**
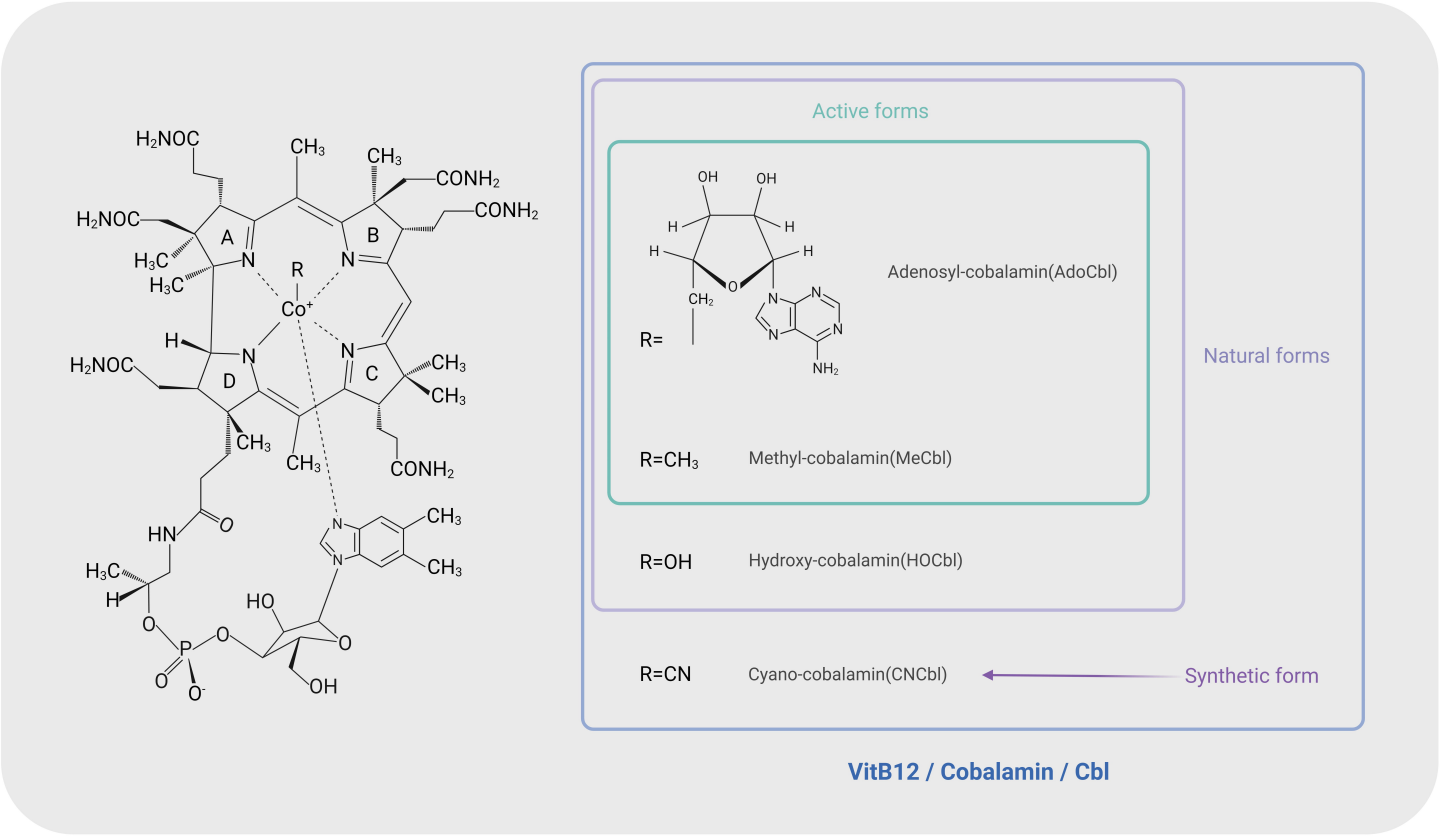
**Different forms of Vitamin B12 (VitB12) and conceptual relationships**. VitB12, also called cobalamin, is a family of structurally related vitamers, including adenosyl-cobalamin (AdoCbl), methyl-cobalamin (MeCbl), hydroxyl-cobalamin (HOCbl), and cyano-cobalamin (CNCbl). Among these compounds, AdoCbl, MeCbl, and HOCbl are natural forms, while CNCbl is a synthetic form and will be transformed into other three forms in the human body. AdoCbl and MeCbl are biologically active coenzyme forms for human metabolic processes [2, 3]. In contrast, pseudo-cobalamin or cobalamin analogs refer to molecules that retain the corrin ring structure but lack bioactivity for humans. These analogs may nevertheless interact within the gut microenvironment, influencing gut microbiota composition and short-chain fatty acids metabolism [4]. The figure was created using BioRender (https://www.biorender.com/).

Vitamin B12 (VitB12), also known as cobalamin (Fig. 1, Ref. [2, 3, 4]), plays a critical role in modulating neurodevelopment [5] and the modulation of psychiatric symptoms, including depression, irritability, hallucinations, and agitation [6]. Within the body, VitB12 exists in two distinct physiological pools: (1) a water-soluble form in the systemic circulation, derived from dietary sources; and (2) components in the gut-brain axis (GBA), which originate from both diet and gut microbiota. Systemic circulation serves as the primary pathway for transporting VitB12 from ileal enterocytes (the site of absorption) to target cells. In target cells, VitB12 is essential for the conversion of homocysteine (Hcy) to methionine, methylmalonyl-CoA to succinyl-CoA [7], and intimately links to the metabolism of one-carbon units, fatty acids [2, 8], amino acids, neurotransmitters [9], myelin synthesis, and DNA/RNA production [10]. Beyond systemic effects, the GBA is a bidirectional communication network connecting gut microbiota and brain function through neuronal, endocrine, and immune pathways [11]. This axis has garnered increasing attention as both a pathophylogical contributor and a therapeutic target for neuropsychiatric disorders, including MDD, BD, schizophrenia, and anxiety [12].

A range of GBA-targeted interventions has been proposed for managing psychiatric disorders, including affective disorders [12]. For instance, probiotic and prebiotic supplementation may restore gut microbiota homeostasis, modulate microbial-derived metabolites (e.g., short-chain fatty acids), and enhance gut barrier integrity [13]. Transcutaneous vagus nerve stimulation has demonstrated potential to improve depressive symptoms by attenuating sympathetic nervous system hyperactivity and reducing systemic inflammatory mediators, thereby enhancing treatment outcomes [14]. Furthermore, emerging evidence suggests that nutritional interventions, particularly VitB12 supplementation, may confer benefits within the GBA framework, potentially addressing neuroinflammation and neurotransmitter dysregulation implicated in mood disorders [15]. However, several issues should be addressed before the clinical application of VitB12 supplementation for affective disorders. For example, the precise mechanisms by which VitB12 improves mood status, the dosage and frequency of VitB12 supplementation, and the potential adverse events caused by VitB12 supplementation. In this review, we explore the multifaceted roles of VitB12 within GBA and discuss its potential as therapeutic adjunct in the management of affective disorders.

## 2. Absorption, Transport and Utilization of VitB12

**Fig. 2.**
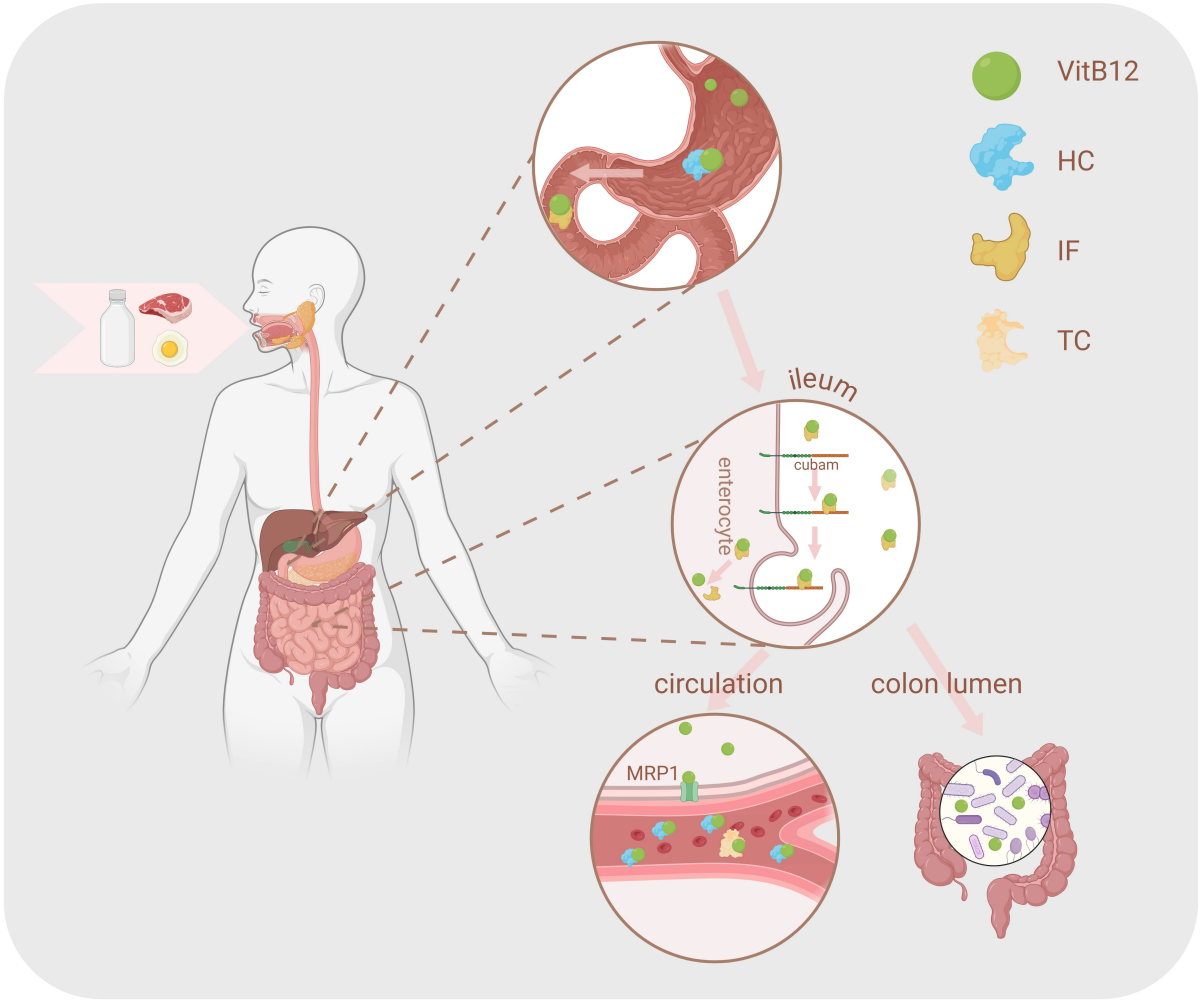
**Absorption and utilization of VitB12**. Food-derived VitB12 is absorbed in the small instestine, with partially entering systemic circulation at the end of the ileum, while the remainder contributes to the gut luminal environment. HC, haptocorrin; IF, intrinsic factor; TC, transcobalamin; MRP1, multidrug resistance protein 1. The figure was created using BioRender (https://www.biorender.com/).

VitB12 is synthesized exclusively by certain bacteria and archaea [2, 16]. While non-human mammals acquire VitB12 through coprophagic behavior or via fermentation processes of ruminant digestive systems [17], human rely entirely on animal-derived foods for VitB12 intake, such as meat, eggs, and dairy [18]. The absorption of VitB12 in humans occurs in a multi-step pathway in the gastrointestinal tract (Fig. 2). In the upper gastrointestinal tract, VitB12 is enzymatically released from dietary proteins and binds to haptocorrin (HC), a glycoprotein presents in saliva and gastric secretions. Upon reaching the duodenum, pancreatic proteases degrade HC, allowing VitB12 to bind to intrinsic factor (IF) and forming the IF-VitB12 complex, which is subsequently internalized by enterocytes in the terminal ileum via the cubilin-amnionless receptor complex (cubam) [19]. Once inside ileal enterocytes, the IF-VitB12 complex dissociates, and free VitB12 is released into systemic circulation through the ATP-binding cassette transporter multidrug resistance protein 1 (MRP1) [7]. In circulation, VitB12 is transported by two binding proteins, HC and transcobalamin (TC). Approximately 80–90% of VitB12 is bound to HC and serves as a storage reservoir, while the remaining 10–20% is bound to TC and is delivered to target tissues, including the liver, via receptor-mediated endocytosis [20]. The TC-VitB12 complex is recognized by receptors such as megalin, cubilin, and the CD320 molecule (CD320) [21, 22, 23]. CD320, in particular, is critical for mediating the uptake of TC-VitB12 by central nervous cells, ensuring its role in neurological function [24].

The human body maintains sufficient VitB12 stores to meet physiological needs for 3 to 5 years [25]. Nearly 50% of this reserve is localized in the liver and maintained through enterohepatic circulation [26]. Additionally, the kidneys also contribute to VitB12 homeostasis through a dual role by secreting excess VitB12 via urine and simultaneously serving as a secondary storage site [27, 28]. This balance is achieved via a filtration-reabsorption mechanism in the renal tubules. In high-income countries, VitB12 deficiency is relatively uncommon in the general adult population due to efficient physiological regulation and widespread nutritional adequacy [29]. However, specific risk factors persist, including food-cobalamin malabsorption [30], inadequate dietary intake (especially in strict vegetarians), elevated physiological demands (pregnant or lactating women), and medications such as metformin and proton pump inhibitors [31].

## 3. VitB12 and Affective Disorders: The Role of Systemic Circulation

### 3.1 Clinical Implications

Several clinical studies have suggested that low serum VitB12 levels and high Hcy concentrations are associated with affective disorders in both adolescents and adults [32, 33, 34, 35, 36, 37, 38, 39], supporting the hypothesis that VitB12 deficiency may lead to Hcy accumulation [7]. Individuals with depression have been found to exhibit significantly higher serum Hcy levels compared to healthy controls, and hyperhomocysteinemia (defined as serum Hcy level >15 µmol/L) has been linked to an increased risk of developing depressive symptoms [32]. In patients with BD, Hcy levels may serve as a potential predictor of mood states, with elevated Hcy levels observed during manic and euthymic phases compared to healthy individuals. Notably, Hcy levels peaked during manic episodes, correlating with greater symptom severity [38]. Furthermore, elevated Hcy levels were linked to poorer clinical outcomes, including higher rates of mixed affective states [33], increased relapse risk, and pronounced cognitive deficits [37]. Collectively, these findings underscore the potential role of Hcy in both the pathophysiology and progression of mood disorders.

Despite growing evidence, the causal relationship between serum VitB12 levels and affective disorders remains unclear. It has been hypothesized that low VitB12 and elevated Hcy levels may contribute to the onset of affective symptoms. Alternatively, the progress of affective disorder and associated pharmacological treatments may negatively impact dietary intake or intestinal absorption of VitB12, leading to secondary deficiency [35]. Sex- and age-related variations further complicate this relationship. For instance, men generally present with higher Hcy levels than women, possibly due to estrogen’s regulatory effects on Hcy metabolism [40, 41]. Observational studies also indicate that deficiencies in both VitB12 and folate contribute to elevated Hcy levels, suggesting that VitB12 insufficiency alone may not fully explain the risk of mood disorders. Mechanistically, VitB12 serves as a coenzyme for methionine synthase, an enzyme essential for the remethylation of Hcy to methionine using 5-methyltetrahydrofolate as a methyl donor. Thus, both VitB12 and folate deficiencies would hamper this process, leading to the accumulation of Hcy and subsequent disruptions in neurotransmitter synthesis, DNA methylation, myelin maintenance, and other processes critical to neurological and mental health [42].

Epidemiological data suggest that approximately 5% of general psychiatric inpatients present with low serum VitB12 levels, with this figure rising to 10–20% among elderly individuals. Notably, chronic VitB12 deficiency spanning over 40 years may contribute to the development of Alzheimer’s disease or multiple sclerosis, suggesting that the neurological consequences of VitB12 deficiency may develop progressively over long-term exposure [43]. Furthermore, subclinical VitB12 deficiency (blood level less than 200 µmol/L), defined as a state of inadequate VitB12 in the absence of overt clinical symptoms or with only mild symptoms [44], has also been implicated in affective disorders. These findings suggest that even mild or asymptomatic deficiencies could induce subtle physiological impairments, potentially influencing mental health outcomes. Nevertheless, the mechanisms by which subclinical VitB12 deficiency contributes to affective disorders, as well as its broader systemic effects, call for more comprehensive investigation.

### 3.2 Neuroprotective and Anti-Inflammatory Effects of VitB12

Emerging evidence reveals neuroinflammation as a critical mediator of neural impairment in mood disorders [45]. Chronic neuroinflammatory processes, driven by dysregulated immune signaling, disrupt synaptic plasticity, impair neurogenesis, and damage neuronal networks, particularly in brain regions like the prefrontal cortex, hippocampus, and amygdala, which govern emotional regulation and cognitive function [45]. Activated microglia,as well as pro-inflammatory cytokines (e.g., IL-6, TNF-α), participate in perpetuating oxidative stress, mitochondrial dysfunction, and excitotoxicity, further compromising neuronal integrity [46]. Notably, sustained neuroinflammation correlates with treatment resistance and core symptoms of mood disorders, including anhedonia and cognitive deficits [47]. While anti-inflammatory therapies show promise in preclinical models, the bidirectional relationship between neuroinflammation and neural dysfunction, as well as its clinical implications, remains an area highlighting the need for more investigations.

VitB12 exerts neuroprotective effects on neural tissues through modulating DNA methylation and bioactive factors metabolism [48, 49, 50]. For instance, de Queiroz *et al*. [48] revealed that VitB12 counteracted hippocampal neuronal damage in a rat model of bacterial meningitis by modulating DNA methylation, stabilizing genomic integrity, and upregulating anti-inflammatory gene expression. S-adenosyl-l-methionine (SAMe), a methyl donor engaging in DNA methylation for genomic instability [48], is not only synthesized systemically through VitB12-engaged one-carbon metabolism [51] but also is derived from gut microbiota in the intestinal lumen [52]. Notably, SAMe itself exhibits antidepressant-like properties via promoting serotonin synthesis and activating the serotonin 1A receptor activation [53]. Similarly, Suryavanshi *et al*. [50] reported that VitB12 supplementation in diabetic models reduced neuronal apoptosis and restored levels of brain-derived neurotrophic factor (BDNF), a key mediator of nerve development, survival, and synaptic plasticity [54]. Reduced levels of BDNF were strongly linked to depression, and its upregulation was a recognized mechanism of antidepressant treatment [54, 55]. Collectively, this evidence highlights the potential of VitB12 in enhancing resilience against affective disorders by preserving neuronal stability through epigenetic, anti-inflammatory, and neurotrophic pathways.

VitB12 alleviates neuroinflammation through multiple mechanisms. For example, Jonnalagadda *et al*. [22] reported that neuroinflammation suppressed the VitB12-transcobalamin 2-CD320 pathway in an animal model of multiple sclerosis, and fingolimod, a therapeutic agent for multiple sclerosis, exerted its anti-inflammatory effects via restoring this pathway. Cassiano *et al*. [56] uncovered that VitB12 attenuated inflammatory infiltrate in the central nervous system, thereby mitigating hippocampal damage and preventing neurological dysfunction in pneumococcal meningitis [57]. These findings collectively implied that the VitB12-mediated modulation of neuroinflammatory processes in the central nervous system was intricately linked to the pathophysiology of neuropsychiatric diseases. In addition, peripheral inflammation alone can trigger functional and structural dysconnectivity in frontostriatal, amygdala-prefrontal, and interoceptive circuits. Connectomic analysis showed that dysregulation within these neuronal dynamic networks underlay mood fluctuations, a core clinical manifestation of BD [58]. Systemic inflammation further disrupted the blood-brain barrier (BBB) and up-regulated reactive oxygen species (ROS), thus creating a neurotoxic milieu that exacerbates affective disorders [57, 59, 60, 61]. This also implied the potential of peripheral inflammatory profiles as predictive biomarkers for affective disorders.

Taken together, VitB12 deficiency leads to the accumulation of ROS, neuroinflammation, and demyelination, contributing to a vicious circle in the central nervous system [57] and potentially involving in the pathogenesis of neuropsychiatric disorders. Conversely, VitB12 supplementation demonstrates neuroprotective properties by mitigating neuronal damage, stabilizing neural integrity, and suppressing inflammatory pathways. However, clinical evidence is lacking to validate this assumption, especially in patients with affective disorders.

## 4. The Gut-Brain Axis: A Microbial Pathway Linking VitB12 and Affective Disorders

### 4.1 How Does VitB12 Affect Gut Microbial Composition and Function?

The impact of dietary derived VitB12 on the gut microbiota remains a subject of debate, owing to inconsistent findings across *in vitro* and *in vivo* studies. *In vitro* experiments suggested that VitB12 supplementation enhanced α-diversity (a measure of microbial community richness and functional redundancy) and altered β-diversity (an indicator of shifts in gut microbiome composition). It also promoted the growth of specific bacteria taxa and boosted the production of short-chain fatty acids (SCFAs). However, these findings have not been consistently replicated in animal studies or population-based research, with variations observed in microbial composition at both phylum and genus levels and no consensus on overarching patterns. Discrepancies may arise from differences in VitB12 formulation, dosage, interactions with co-administered nutrients or medications, and host-specific variables [8, 62, 63]. Recent studies in *Caenorhabditis elegans* [9, 64] and zebrafish [16] have illuminated the role of VitB12 in modulating the GBA, affecting balanced gut microbial networks and cholinergic signalling. In humans, Oliphant *et al*. [5] found that gut microbial function in VitB12 synthesis was positively associated with cognitive performance and stress regulation in infants, suggesting its critical role in neurodevelopment. Given that the gut microbiome stabilizes by around three years of age [65], further research is warranted to explore the benefits of VitB12 intervention in infants and pregnant women, potentially harnessing early developmental plasticity to strengthen resilience against affective disorders later in life.

Within the gut microenvironment, VitB12 serves as an essential nutrient for microbial communities. About 20% of gut bacteria synthesize VitB12 *de novo*, while 80% possess receptors for absorbing VitB12, highlighting its important role in microbial survival and cooperation [66]. There are two major routes (aerobic and anaerobic) by certain gut microbes to synthesize VitB12 *de novo*. The two routes diverge in their cobalt chelation mechanisms and oxygen dependencies, as the aerobic pathway requires oxygen to catalyze ring-contraction, whereas the anaerobic pathway proceeds independently of oxygen [67]. VitB12 is involved in cross-feeding interactions in microbe-microbe and microbe-host patterns [68], and microbe-derived VitB12 is self-sufficient in healthy adult gut [69]. For *Escherichia coli* , the presence of VitB12 suppresses the expression of *BtuB*, a gene encoding a transportor responsible for active VitB12 intake [70]. This feedback mechanism illustrates how commensal bacteria dynamically regulate VitB12 utilization to adapt to its availability, optimizing metabolic efficiency within the gut ecosystem.

VitB12 supplementation has limited effects on the gut environment in both *in vitro* and *in vivo* studies [8, 69], suggesting the existence of regulatory mechanisms that stabilize VitB12 levels in the gut lumen. For example, Kelly *et al*. [71] research indicated that high-dose VitB12 supplementation suppressed the endogenous VitB12 production by gut microbiota, suggesting that gut microbiota can sense VitB12 concentrations and regulate its production. Similarly, Li *et al*. [72] confirmed that in *Propionibacterium* strain UF1, a high dose (750 µM) of VitB12 completely inhibited gene expression responsible for VitB12 biosynthesis. These findings highlight how VitB12-dependent microbiota and their metabolic activities are integrated into a homeostatic system. Notably, while VitB12 supplementation depleted *Bacteroides* populations, it failed to change SCFAs concentration or microbial diversity in the distal gut, despite depleting, implying the intrinsic resilience in the gut lumen [71]. Further complexity arises from interactions between gut microbiota and corrinoids, a class of compounds including cobalamin and cobalamin analogs. High-dose (exceeding the recommended levels by 1000-fold) VitB12 supplementation temporarily disrupted corrinoid profiles, but it returned to the baseline level within 10 days [4, 73]. Degnan *et al*. [73] even proposed an inspiring idea to use non-metabolizable corrinoids that can evade microbial remodelling mechanisms to manipulate the composition and function of gut microbiota. However, major questions remain regarding how microbes discriminate among corrinoids, the dynamics of cobalamin-to-analog conversion, and the precise molecular mechanisms that govern microbial remodeling processes. Altogether, these findings challenge the assumption that dietary VitB12 supplementation has a straightforward or broadly beneficial impact on gut microbiota. Instead, they underscore the complexity and resilience of microbial ecosystems and highlight the need for deeper investigation into how VitB12 and related compounds functionally reshape gut microbial ecology and, by extension, influence mental health outcomes.

### 4.2 VitB12 Interacts With Gut-Brain Axis and Affective Disorders

Clinical evidence highlights the role of VitB12 in mediating the interactions between GBA and affective disorders. Both dietary and gut microbiota-derived VitB12 help to keep a complex, balanced, and stable gut microbial network [9, 16]. Studies consistently indicated discrepancies in gut microbiota composition, stability index, and disrupted metabolite profiles in individuals with affective disorders compared to healthy controls. For example, in patients with MDD, fecal samples mostly showed decreased α-diversity, increased relative abundance of *Actinobacteria* (phylum), *Bifidobacteriaceae* (family), and *Bacteroides* (genus), alongside decreased abundance of *Ruminococcaceae* (family), *Faecalibacterium*, and *Roseburia* (genus) [74]. Strikingly, fecal microbiota transplantation from MDD patients to germ-free mice can induce depression-like behaviors [75], while antidepressant (R)-ketamine treatment converted the gut microbial dysbiosis and behavioral deficits [76]. Similarly, unpredictable chronic mild stress in rats led to depressive-like behaviors and altered metabolomic profiles in the hippocampus and jejunum [77], further implicating gut-brain crosstalk. Likewise, *Bacteroidetes* was the predominant gut phylum in BD patients, while *Firmicutes* predominated in healthy controls, and quetiapine treatment can alter gut microbial composition in patients with BD [78, 79, 80]. Moreover, serum metabolomic analysis in BD patients revealed distinct signatures compared to healthy controls, with gut microbe-derived “neuroactive metabolites” correlating with symptom severity [81]. These findings implicate VitB12 as a potential modulator of GBA dysfunction in affective disorders, linking microbial stability and host metabolic activities.

VitB12 plays a significant role in modulating inflammation implicated in MDD and BD [82, 83]. It activates gene expression in ileal epithelial cells by sustaining cellular methylation programs, thereby stimulating cell proliferation, enhancing SCFAs metabolism, and suppressing pro-inflammatory pathways [51]. SCFAs (e.g., acetate, propionate, and butyrate) are produced by gut microbiota through the fermentation of dietary fibres [84]. These metabolites can strengthen gut mucosal barrier [85], exert anti-inflammatory effects [12, 51], maintain enteric serotonin production [86], up-regulate BDNF [87], and reduce ROS accumulation [88]. In addition to dietary VitB12 [69], microbe-derived pseudo-VitB12 can promote propionate production independent of diet [89]. Meanwhile, butyrate, propionate, as well as VitB12 itself, serve as protectors to the gut barrier [16, 51, 89]. Food-derived SCFAs can normalize the immunodeficiency of germ-free mice via promoting microglia maturation, suggesting their role in maintaining immune homeostasis [90, 91]. Furthermore, SCFAs can cross the gut-blood barrier and exert an anti-inflammatory effect via binding to G-protein-coupled receptor 43 in systematic circulation [92]. Microbe-derived propionate protects BBB against ROS-induced damage [93]. Moreover, emerging research found that the vagus nerve acted as a key regulator in the gut-brain axis, bidirectionally mediating inflammatory signals between the brain and peripheral immune response [94]. The vagus nerve also transmits brain stress signals to regulate gut microbiota and immune activity [95]. This intricate crosstalk positions VitB12 and SCFAs as critical regulators of neuroimmune interactions in affective disorders.

**Fig. 3.**
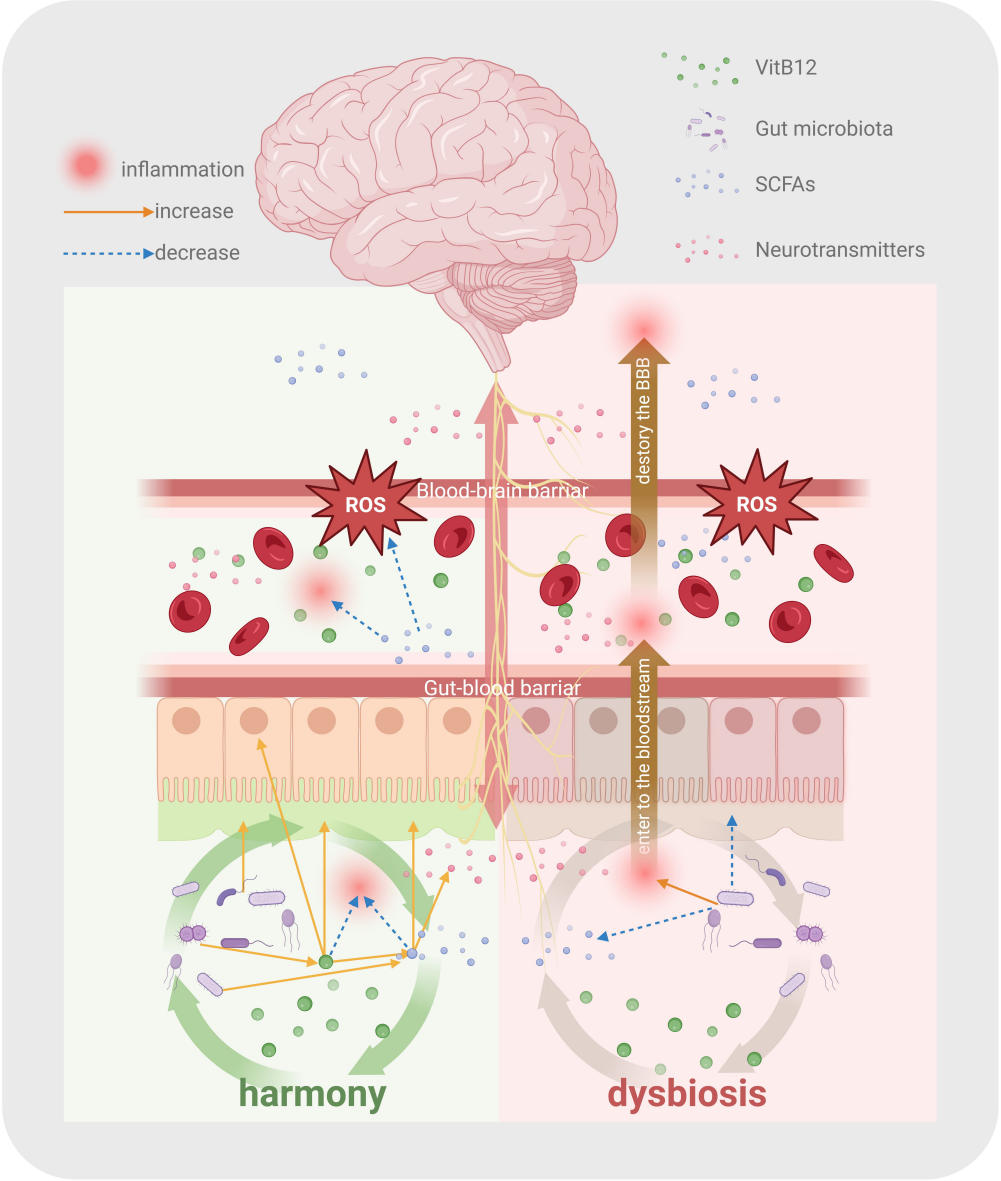
**VitB12 modulates the functional equilibrium of the gut-brain axis**. Under homeostatic conditions, VitB12 interacts with gut mucosa, microbiota, and microbial metabolites, synergistically suppressing inflammation and maintaining homeostasis of the gut-brain axis. Under dysbiosis states, these complex interactions are disrupted, triggering inflammatory cascades in both peripheral circulation and central nervous system, thereby increasing susceptibility to affective disorders. SCFAs, short-chain fatty acids; BBB, the blood-brain barrier; ROS, reactive oxygen species. The figure was created using BioRender (https://www.biorender.com/).

Under pathological conditions of affective disorders, gut microbial composition exhibits a transdiagnostic pattern, characterized by a reduction in the abundance of butyrate-producing bacteria with anti-inflammatory properties and an increase in pro-inflammatory genera [12, 96]. Disturbance of the gut microbiome disrupts the permeability of the intestinal barrier, allowing the translocation of inflammatory molecules, bacterial endotoxins, and neuroactive metabolites into systemic circulation. Through bidirectional gut-brain crosstalk [81], these mediators propagate chronic systemic inflammation, which compromises the BBB and drives neuroinflammation [97, 98]. Collectively, VitB12, gut microbiota, SCFAs, gut mucosal defenses, and vagal neurotransmission interact synergistically within the GBA to maintain gut and neuroimmune homeostasis and enhance resilience to affective disorders (Fig. 3).

## 5. VitB12 Supplementation as a Therapeutic Strategy in Affective Disorders

For patients diagnosed with VitB12 deficiency, treatment recommendations vary based on symptom severity and absorption capacity. Those presenting with acute symptoms or malabsorption are recommended to receive intramuscular VitB12 injections, while those with no or mild symptoms and without absorption or compliance concerns may be better suited for oral supplementation [29]. Oral supplements showed equal efficacy to intramuscular injection in VitB12 deficiency related to gastrointestinal disorders [99, 100], supporting the feasibility of oral administration in such cases. As for VitB12 forms, Xu *et al*. [101] found that despite methylcobalamin (MeCbl) and hydroxylcobalamin (HOCbl) exhibited equivalent bioavailability, MeCbl was more favorable for gut microbiome and microbial metabolism, suggesting MeCbl is the preferred choice for oral supplementation. As a water-soluble vitamin, excess VitB12 can be excreted renally, rendering it generally non-toxic even in cases of overdose. Nonetheless, it is preferred that VitB12 should be supplied within an appropriate dosage. Intestinal absorption efficacy declines sharply with higher doses, dropping from approximately 50% for dose below 50 µg to just 1% at 500 µg [3]. Similarly, cellular absorption dose not improve with supraphysiological concentrations [102]. A case report documented VitB12 overdose triggered mixed episodes of BD in a susceptible individual [103]. This also enlightens that VitB12 supplementation should be within a reasonable dosage to mitigate potential risks.

To date, no consensus has been reached regarding the use of VitB12 supplementation in patients with affective disorders. Higher VitB12 level has been proven to relate to better ketamine efficacy [104]. Further studies and clinical trials are therefore needed to explore whether VitB12 supplementation contributes to clinical drug efficacy and disease prognosis.

## 6. Limitations and Perspectives

Whether microbe-derived VitB12 can be absorbed into the bloodstream via the colon requires further exploration. In traditional perspective, microbe-derived VitB12 is unavailable for human consumption, as absorption primarily occurs in the ileum, a site upstream of the predominant colonization of gut microbiota. However, Sun *et al*. [105] reported an intriguing exception that microbe-derived VitB12 can enter systemic circulation, evidenced by elevated serum VitB12 levels correlating with fecal concentrations. Nevertheless, this interpretation may be confounded by the fact that the liver was the primary storage organ for VitB12, and Silymarin’s effects on liver lipid metabolism may result in alterations to plasma VitB12 concentrations. Previous evidence indicated that small intestinal bacterial overgrowth may lead to VitB12 loss due to host-microbe competition in the small intestine [73, 106]. Whereas bacterial density in the healthy small intestine remains below than 10^5^ CFU/mL [107], the composition and function of these intestinal bacteria are ongoingly being investigated.

Although VitB12 is the focus of this review, a variety of nutritional supplementation is increasingly used as adjunct treatments in patients with affective disorders [108]. The interplay and collaborative effects of B vitamins warrant further investigations. For instance, Rakić *et al*. [109] proved that a vitamin B complex (VBC, composed of B1, B2, B3, B5, B6, and B12) exerted neuroprotective effects by alleviating neuroinflammation. The same team also found that VBC can repair peripheral nerve cells and improve gut microbiota dysbiosis due to autoimmune encephalomyelitis [110]. Notably, synergistic effects of vitamin B1, B6, and B12 surpass the efficacy of any single B vitamin, implying the potential of VBC in treating peripheral neuropathy [10, 111]. Moreover, vitamin B (folate, B2, B6, and B12) may contribute to the production of butyrate, as vitamin B supplementation has been linked to the increased relative abundance of butyrate-producing commensal bacteria. This suggests a collaborative role of B vitamins in enhancing microenvironmental homeostasis [112].

## 7. Conclusion

In summary, VitB12 functions not as an isolated regulator but as an integral component of the GBA system, influencing gut microbial ecology, neuroimmune homeostasis, and host metabolic signalling. Rather than acting independently, VitB12 interacts with microbial communities, short-chain fatty acids, and neuroinflammatory pathways to shape affective outcomes. This positions VitB12 as a promising avenue for advancing our understanding of the pathophysiology of affective disorders and for identifying novel adjunctive therapeutic strategies. To establish its clinical relevance, further human-based studies are needed to elucidate the underlying mechanism of VitB12 to gut-brain communication and to evaluate its clinical applicability as a complementary intervention within the GBA framework.

## Abbreviations

VitB12, Vitamin B12; GBA, the gut-brain axis; MDD, major depressive disorder; BD, bipolar disorder; Hcy, homocysteine; AdoCbl, adenosyl-cobalamin; MeCbl, methyl-cobalamin; HOCbl, hydroxyl-cobalamin; CNCbl, cyano-cobalamin; SCFAs, short-chain fatty acids; HC, haptocorrin; IF, intrinsic factor; MRP1, multidrug resistance protein 1; CD320, CD320 molecule; TC, transcobalamin; SAMe, S-adenosyl-l-methionine; BDNF, brain-derived neurotrophic factor; BBB, the blood-brain barrier; ROS, reactive oxygen species; VBC, vitamin B complex.

## References

[1] GBD 2019 Mental Disorders Collaborators (2022). Global, regional, and national burden of 12 mental disorders in 204 countries and territories, 1990-2019: a systematic analysis for the Global Burden of Disease Study 2019. *The Lancet. Psychiatry*.

[2] Martens JH, Barg H, Warren MJ, Jahn D (2002). Microbial production of vitamin B12. *Applied Microbiology and Biotechnology*.

[3] Paul C, Brady DM (2017). Comparative Bioavailability and Utilization of Particular Forms of B12 Supplements With Potential to Mitigate B12-related Genetic Polymorphisms. *Integrative Medicine*.

[4] Allen RH, Stabler SP (2008). Identification and quantitation of cobalamin and cobalamin analogues in human feces. *The American Journal of Clinical Nutrition*.

[5] Oliphant K, Cruz Ayala W, Ilyumzhinova R, Mbayiwa K, Sroka A, Xie B (2024). Microbiome function and neurodevelopment in Black infants: vitamin B12 emerges as a key factor. *Gut Microbes*.

[6] Lindenbaum J, Healton EB, Savage DG, Brust JC, Garrett TJ, Podell ER (1988). Neuropsychiatric disorders caused by cobalamin deficiency in the absence of anemia or macrocytosis. *New England Journal of Medicine*.

[7] Nielsen MJ, Rasmussen MR, Andersen CBF, Nexø E, Moestrup SK (2012). Vitamin B12 transport from food to the body’s cells–a sophisticated, multistep pathway. *Nature Reviews. Gastroenterology & Hepatology*.

[8] Guetterman HM, Huey SL, Knight R, Fox AM, Mehta S, Finkelstein JL (2022). Vitamin B-12 and the Gastrointestinal Microbiome: A Systematic Review. *Advances in Nutrition*.

[9] Zhu X, Xia Y, Wang H, Shi L, Yin H, Gu M (2023). PM2.5 induced neurotoxicity through unbalancing vitamin B12 metabolism by gut microbiota disturbance. *Gut Microbes*.

[10] Calderón-Ospina CA, Nava-Mesa MO (2020). B Vitamins in the nervous system: Current knowledge of the biochemical modes of action and synergies of thiamine, pyridoxine, and cobalamin. *CNS Neuroscience & Therapeutics*.

[11] Młynarska E, Gadzinowska J, Tokarek J, Forycka J, Szuman A, Franczyk B (2022). The Role of the Microbiome-Brain-Gut Axis in the Pathogenesis of Depressive Disorder. *Nutrients*.

[12] Nikolova VL, Smith MRB, Hall LJ, Cleare AJ, Stone JM, Young AH (2021). Perturbations in Gut Microbiota Composition in Psychiatric Disorders: A Review and Meta-analysis. *JAMA Psychiatry*.

[13] Ribera C, Sánchez-Ortí JV, Clarke G, Marx W, Mörkl S, Balanzá-Martínez V (2024). Probiotic, prebiotic, synbiotic and fermented food supplementation in psychiatric disorders: A systematic review of clinical trials. *Neuroscience and Biobehavioral Reviews*.

[14] Liu CH, Yang MH, Zhang GZ, Wang XX, Li B, Li M (2020). Neural networks and the anti-inflammatory effect of transcutaneous auricular vagus nerve stimulation in depression. *Journal of Neuroinflammation*.

[15] Cicero AF, Minervino A (2022). Combined action of SAMe, Folate, and Vitamin B12 in the treatment of mood disorders: a review. *European Review for Medical and Pharmacological Sciences*.

[16] Qi X, Zhang Y, Zhang Y, Luo F, Song K, Wang G (2023). Vitamin B12 produced by Cetobacterium somerae improves host resistance against pathogen infection through strengthening the interactions within gut microbiota. *Microbiome*.

[17] Kennedy DO (2016). B Vitamins and the Brain: Mechanisms, Dose and Efficacy–A Review. *Nutrients*.

[18] Wolffenbuttel BH, Owen PJ, Ward M, Green R (2023). Vitamin B12.

[19] Fyfe JC, Madsen M, Højrup P, Christensen EI, Tanner SM, de la Chapelle A (2004). The functional cobalamin (vitamin B12)-intrinsic factor receptor is a novel complex of cubilin and amnionless. *Blood*.

[20] McCorvie TJ, Ferreira D, Yue WW, Froese DS (2023). The complex machinery of human cobalamin metabolism. *Journal of Inherited Metabolic Disease*.

[21] Quadros EV, Nakayama Y, Sequeira JM (2009). The protein and the gene encoding the receptor for the cellular uptake of transcobalamin-bound cobalamin. *Blood*.

[22] Jonnalagadda D, Kihara Y, Groves A, Ray M, Saha A, Ellington C (2023). FTY720 requires vitamin B12-TCN2-CD320 signaling in astrocytes to reduce disease in an animal model of multiple sclerosis. *Cell Reports*.

[23] Kozyraki R, Cases O (2013). Vitamin B12 absorption: mammalian physiology and acquired and inherited disorders. *Biochimie*.

[24] Alam A, Woo JS, Schmitz J, Prinz B, Root K, Chen F (2016). Structural basis of transcobalamin recognition by human CD320 receptor. *Nature Communications*.

[25] Danchin A, Braham S (2017). Coenzyme B12 synthesis as a baseline to study metabolite contribution of animal microbiota. *Microbial Biotechnology*.

[26] Carmel R (2013). Diagnosis and management of clinical and subclinical cobalamin deficiencies: why controversies persist in the age of sensitive metabolic testing. *Biochimie*.

[27] Birn H (2006). The kidney in vitamin B12 and folate homeostasis: characterization of receptors for tubular uptake of vitamins and carrier proteins. *American Journal of Physiology. Renal Physiology*.

[28] Birn H, Willnow TE, Nielsen R, Norden AGW, Bönsch C, Moestrup SK (2002). Megalin is essential for renal proximal tubule reabsorption and accumulation of transcobalamin-B(12). *American Journal of Physiology. Renal Physiology*.

[29] Langan RC, Goodbred AJ (2017). Vitamin B12 Deficiency: Recognition and Management. *American Family Physician*.

[30] Andrès E, Perrin AE, Demangeat C, Kurtz JE, Vinzio S, Grunenberger F (2003). The syndrome of food-cobalamin malabsorption revisited in a department of internal medicine. A monocentric cohort study of 80 patients. *European Journal of Internal Medicine*.

[31] Hunt A, Harrington D, Robinson S (2014). Vitamin B12 deficiency. *BMJ (Clinical Research Ed.)*.

[32] Moradi F, Lotfi K, Armin M, Clark CCT, Askari G, Rouhani MH (2021). The association between serum homocysteine and depression: A systematic review and meta-analysis of observational studies. *European Journal of Clinical Investigation*.

[33] Ozdogan MG, Aydin EF, Ustundag MF, Ceyhun HA, Oral E, Bakan E (2020). Homocysteine, chronotype and clinical course in bipolar disorder patients. *Nordic Journal of Psychiatry*.

[34] Esnafoglu E, Ozturan DD (2020). The relationship of severity of depression with homocysteine, folate, vitamin B12, and vitamin D levels in children and adolescents. *Child and Adolescent Mental Health*.

[35] Khosravi M, Sotoudeh G, Amini M, Raisi F, Mansoori A, Hosseinzadeh M (2020). The relationship between dietary patterns and depression mediated by serum levels of Folate and vitamin B12. *BMC Psychiatry*.

[36] Zhou SJ, Zhang LG, Chen HM, Li JY, Li R, Zhang XM (2018). Prevalence and clinical-demographic correlates of hyperhomocysteinemia in inpatients with bipolar disorder in a Han Chinese population. *Psychiatry Research*.

[37] Chen PH, Liu HC, Lu ML, Chen CH, Chang CJ, Chiu WC (2019). Homocysteine, rather than age of onset, is a better predictor for cognitive function in older adults with bipolar disorder. *International Journal of Geriatric Psychiatry*.

[38] Salagre E, Vizuete AF, Leite M, Brownstein DJ, McGuinness A, Jacka F (2017). Homocysteine as a peripheral biomarker in bipolar disorder: A meta-analysis. *European Psychiatry: the Journal of the Association of European Psychiatrists*.

[39] Wang C, Lv L, Xin B, Li N, Wang J, An C (2024). Study on the Correlation between Hcy and Hs-CRP Levels and Cognitive Function in Patients with Bipolar Disorder and Borderline Personality Disorder. *Actas Espanolas De Psiquiatria*.

[40] Morris MS, Jacques PF, Selhub J, Rosenberg IH (2000). Total homocysteine and estrogen status indicators in the Third National Health and Nutrition Examination Survey. *American Journal of Epidemiology*.

[41] Margalit I, Cohen E, Goldberg E, Krause I (2018). Vitamin B12 Deficiency and the Role of Gender: A Cross-Sectional Study of a Large Cohort. *Annals of Nutrition & Metabolism*.

[42] Ulloque-Badaracco JR, Hernandez-Bustamante EA, Alarcon-Braga EA, Al-Kassab-Córdova A, Cabrera-Guzmán JC, Herrera-Añazco P (2023). Vitamin B12, folate, and homocysteine in metabolic syndrome: a systematic review and meta-analysis. *Frontiers in Endocrinology*.

[43] Reynolds E (2006). Vitamin B12, folic acid, and the nervous system. *The Lancet. Neurology*.

[44] Hannibal L, Lysne V, Bjørke-Monsen AL, Behringer S, Grünert SC, Spiekerkoetter U (2016). Biomarkers and Algorithms for the Diagnosis of Vitamin B12 Deficiency. *Frontiers in Molecular Biosciences*.

[45] Guo B, Zhang M, Hao W, Wang Y, Zhang T, Liu C (2023). Neuroinflammation mechanisms of neuromodulation therapies for anxiety and depression. *Translational Psychiatry*.

[46] Wang H, He Y, Sun Z, Ren S, Liu M, Wang G (2022). Microglia in depression: an overview of microglia in the pathogenesis and treatment of depression. *Journal of Neuroinflammation*.

[47] Li Q, Xie Y, Lin J, Li M, Gu Z, Xin T (2025). Microglia Sing the Prelude of Neuroinflammation-Associated Depression. *Molecular Neurobiology*.

[48] de Queiroz KB, Cavalcante-Silva V, Lopes FL, Rocha GA, D’Almeida V, Coimbra RS (2020). Vitamin B12 is neuroprotective in experimental pneumococcal meningitis through modulation of hippocampal DNA methylation. *Journal of Neuroinflammation*.

[49] Khiroya K, Sekyere E, McEwen B, Bayes J (2025). Nutritional considerations in major depressive disorder: current evidence and functional testing for clinical practice. *Nutrition Research Reviews*.

[50] Suryavanshi U, Angadi KK, Reddy VS, Reddy GB (2024). Neuroprotective role of vitamin B12 in streptozotocin-induced type 1 diabetic rats. *Chemico-biological Interactions*.

[51] Ge Y, Zadeh M, Mohamadzadeh M (2022). Vitamin B12 Regulates the Transcriptional, Metabolic, and Epigenetic Programing in Human Ileal Epithelial Cells. *Nutrients*.

[52] Tillmann S, Awwad HM, Eskelund AR, Treccani G, Geisel J, Wegener G (2018). Probiotics Affect One-Carbon Metabolites and Catecholamines in a Genetic Rat Model of Depression. *Molecular Nutrition & Food Research*.

[53] Sales AJ, Maciel IS, Crestani CC, Guimarães FS, Joca SR (2023). S-adenosyl-l-methionine antidepressant-like effects involve activation of 5-HT1A receptors. *Neurochemistry International*.

[54] Li Y, Jia Y, Wang D, Zhuang X, Li Y, Guo C (2021). Programmed cell death 4 as an endogenous suppressor of BDNF translation is involved in stress-induced depression. *Molecular Psychiatry*.

[55] Dwivedi Y (2009). Brain-derived neurotrophic factor: role in depression and suicide. *Neuropsychiatric Disease and Treatment*.

[56] Cassiano LMG, Oliveira MDS, de Queiroz KB, Amancio AMTDS, Salim ACDM, Fernandes GDR (2023). Uncovering the neuroprotective effect of vitamin B12 in pneumococcal meningitis: insights into its pleiotropic mode of action at the transcriptional level. *Frontiers in Immunology*.

[57] Mathew AR, Di Matteo G, La Rosa P, Barbati SA, Mannina L, Moreno S (2024). Vitamin B12 Deficiency and the Nervous System: Beyond Metabolic Decompensation-Comparing Biological Models and Gaining New Insights into Molecular and Cellular Mechanisms. *International Journal of Molecular Sciences*.

[58] Perry A, Roberts G, Mitchell PB, Breakspear M (2019). Connectomics of bipolar disorder: a critical review, and evidence for dynamic instabilities within interoceptive networks. *Molecular Psychiatry*.

[59] Goldsmith DR, Bekhbat M, Mehta ND, Felger JC (2023). Inflammation-Related Functional and Structural Dysconnectivity as a Pathway to Psychopathology. *Biological Psychiatry*.

[60] Huppert J, Closhen D, Croxford A, White R, Kulig P, Pietrowski E (2010). Cellular mechanisms of IL-17-induced blood-brain barrier disruption. *FASEB Journal*.

[61] Goldsmith DR, Rapaport MH, Miller BJ (2016). A meta-analysis of blood cytokine network alterations in psychiatric patients: comparisons between schizophrenia, bipolar disorder and depression. *Molecular Psychiatry*.

[62] Albert MJ, Mathan VI, Baker SJ (1980). Vitamin B12 synthesis by human small intestinal bacteria. *Nature*.

[63] He Y, Wu W, Zheng HM, Li P, McDonald D, Sheng HF (2018). Regional variation limits applications of healthy gut microbiome reference ranges and disease models. *Nature Medicine*.

[64] Kang WK, Florman JT, Araya A, Fox BW, Thackeray A, Schroeder FC (2024). Vitamin B12 produced by gut bacteria modulates cholinergic signalling. *Nature Cell Biology*.

[65] Voreades N, Kozil A, Weir TL (2014). Diet and the development of the human intestinal microbiome. *Frontiers in Microbiology*.

[66] Degnan PH, Barry NA, Mok KC, Taga ME, Goodman AL (2014). Human gut microbes use multiple transporters to distinguish vitamin B₁₂ analogs and compete in the gut. *Cell Host & Microbe*.

[67] Fang H, Kang J, Zhang D (2017). Microbial production of vitamin B12: a review and future perspectives. *Microbial Cell Factories*.

[68] Seth EC, Taga ME (2014). Nutrient cross-feeding in the microbial world. *Frontiers in Microbiology*.

[69] Kundra P, Geirnaert A, Pugin B, Morales Martinez P, Lacroix C, Greppi A (2022). Healthy adult gut microbiota sustains its own vitamin B12 requirement in an in vitro batch fermentation model. *Frontiers in Nutrition*.

[70] Lundrigan MD, Köster W, Kadner RJ (1991). Transcribed sequences of the Escherichia coli btuB gene control its expression and regulation by vitamin B12. *Proceedings of the National Academy of Sciences of the United States of America*.

[71] Kelly CJ, Alexeev EE, Farb L, Vickery TW, Zheng L, Eric L C (2019). Oral vitamin B12 supplement is delivered to the distal gut, altering the corrinoid profile and selectively depleting Bacteroides in C57BL/6 mice. *Gut Microbes*.

[72] Li J, Ge Y, Zadeh M, Curtiss R, Mohamadzadeh M (2020). Regulating vitamin B12 biosynthesis via the cbiMCbl riboswitch in Propionibacterium strain UF1. *Proceedings of the National Academy of Sciences of the United States of America*.

[73] Degnan PH, Taga ME, Goodman AL (2014). Vitamin B12 as a modulator of gut microbial ecology. *Cell Metabolism*.

[74] Knuesel T, Mohajeri MH (2021). The Role of the Gut Microbiota in the Development and Progression of Major Depressive and Bipolar Disorder. *Nutrients*.

[75] Zhou M, Fan Y, Xu L, Yu Z, Wang S, Xu H (2023). Microbiome and tryptophan metabolomics analysis in adolescent depression: roles of the gut microbiota in the regulation of tryptophan-derived neurotransmitters and behaviors in human and mice. *Microbiome*.

[76] Hashimoto K (2023). Neuroinflammation through the vagus nerve-dependent gut-microbiota-brain axis in treatment-resistant depression. *Progress in Brain Research*.

[77] Xu Q, Jiang M, Gu S, Zhang X, Feng G, Ma X (2022). Metabolomics changes in brain-gut axis after unpredictable chronic mild stress. *Psychopharmacology*.

[78] Lai J, Jiang J, Zhang P, Xi C, Wu L, Gao X (2020). Gut microbial clues to bipolar disorder: State-of-the-art review of current findings and future directions. *Clinical and Translational Medicine*.

[79] Hu S, Li A, Huang T, Lai J, Li J, Sublette ME (2019). Gut Microbiota Changes in Patients with Bipolar Depression. *Advanced Science*.

[80] Lu Q, Lai J, Lu H, Ng C, Huang T, Zhang H (2019). Gut Microbiota in Bipolar Depression and Its Relationship to Brain Function: An Advanced Exploration. *Frontiers in Psychiatry*.

[81] Li Z, Lai J, Zhang P, Ding J, Jiang J, Liu C (2022). Multi-omics analyses of serum metabolome, gut microbiome and brain function reveal dysregulated microbiota-gut-brain axis in bipolar depression. *Molecular Psychiatry*.

[82] Lai J, Zhang P, Jiang J, Mou T, Li Y, Xi C (2021). New Evidence of Gut Microbiota Involvement in the Neuropathogenesis of Bipolar Depression by TRANK1 Modulation: Joint Clinical and Animal Data. *Frontiers in Immunology*.

[83] Isgren A, Sellgren C, Ekman CJ, Holmén-Larsson J, Blennow K, Zetterberg H (2017). Markers of neuroinflammation and neuronal injury in bipolar disorder: Relation to prospective clinical outcomes. *Brain, Behavior, and Immunity*.

[84] Kasubuchi M, Hasegawa S, Hiramatsu T, Ichimura A, Kimura I (2015). Dietary gut microbial metabolites, short-chain fatty acids, and host metabolic regulation. *Nutrients*.

[85] van de Wouw M, Schellekens H, Dinan TG, Cryan JF (2017). Microbiota-Gut-Brain Axis: Modulator of Host Metabolism and Appetite. *The Journal of Nutrition*.

[86] Reigstad CS, Salmonson CE, Rainey JF, Szurszewski JH, Linden DR, Sonnenburg JL (2015). Gut microbes promote colonic serotonin production through an effect of short-chain fatty acids on enterochromaffin cells. *FASEB Journal*.

[87] Suda K, Matsuda K (2022). How Microbes Affect Depression: Underlying Mechanisms via the Gut-Brain Axis and the Modulating Role of Probiotics. *International Journal of Molecular Sciences*.

[88] Mishra N, Garg A, Ashique S, Bhatt S (2024). Potential of postbiotics for the treatment of metabolic disorders. *Drug Discovery Today*.

[89] Belzer C, Chia LW, Aalvink S, Chamlagain B, Piironen V, Knol J (2017). Microbial Metabolic Networks at the Mucus Layer Lead to Diet-Independent Butyrate and Vitamin B12 Production by Intestinal Symbionts. *mBio*.

[90] Ma Q, Xing C, Long W, Wang HY, Liu Q, Wang RF (2019). Impact of microbiota on central nervous system and neurological diseases: the gut-brain axis. *Journal of Neuroinflammation*.

[91] Erny D, Hrabě de Angelis AL, Jaitin D, Wieghofer P, Staszewski O, David E (2015). Host microbiota constantly control maturation and function of microglia in the CNS. *Nature Neuroscience*.

[92] Maslowski KM, Vieira AT, Ng A, Kranich J, Sierro F, Yu D (2009). Regulation of inflammatory responses by gut microbiota and chemoattractant receptor GPR43. *Nature*.

[93] Hoyles L, Snelling T, Umlai UK, Nicholson JK, Carding SR, Glen RC (2018). Microbiome-host systems interactions: protective effects of propionate upon the blood-brain barrier. *Microbiome*.

[94] Jin H, Li M, Jeong E, Castro-Martinez F, Zuker CS (2024). A body-brain circuit that regulates body inflammatory responses. *Nature*.

[95] Chang H, Perkins MH, Novaes LS, Qian F, Zhang T, Neckel PH (2024). Stress-sensitive neural circuits change the gut microbiome via duodenal glands. *Cell*.

[96] Chen X, Shi S, Sun C, Li S (2024). A Study of the Relationship between Inflammatory Immune Function and Intestinal Flora in Adolescent Patients with First-Episode Depression. *Actas Espanolas De Psiquiatria*.

[97] Parker A, Fonseca S, Carding SR (2020). Gut microbes and metabolites as modulators of blood-brain barrier integrity and brain health. *Gut Microbes*.

[98] Kebir H, Kreymborg K, Ifergan I, Dodelet-Devillers A, Cayrol R, Bernard M (2007). Human TH17 lymphocytes promote blood-brain barrier disruption and central nervous system inflammation. *Nature Medicine*.

[99] Andrès E, Zulfiqar AA, Serraj K, Vogel T, Kaltenbach G (2018). Systematic Review and Pragmatic Clinical Approach to Oral and Nasal Vitamin B12 (Cobalamin) Treatment in Patients with Vitamin B12 Deficiency Related to Gastrointestinal Disorders. *Journal of Clinical Medicine*.

[100] Wang H, Li L, Qin LL, Song Y, Vidal-Alaball J, Liu TH (2018). Oral vitamin B12 versus intramuscular vitamin B12 for vitamin B12 deficiency. *The Cochrane Database of Systematic Reviews*.

[101] Xu Y, Xiang S, Ye K, Zheng Y, Feng X, Zhu X (2018). Cobalamin (Vitamin B12) Induced a Shift in Microbial Composition and Metabolic Activity in an in vitro Colon Simulation. *Frontiers in Microbiology*.

[102] Boachie J, Adaikalakoteswari A, Goljan I, Samavat J, Cagampang FR, Saravanan P (2021). Intracellular and Tissue Levels of Vitamin B12 in Hepatocytes Are Modulated by CD320 Receptor and TCN2 Transporter. *International Journal of Molecular Sciences*.

[103] Stachura A, Banaszek Ł, Jurkin K, Święcicki Ł (2024). Vitamin B12 overdose may trigger the onset of mixed-state bipolar disorder: A case report. *Bipolar Disorders*.

[104] Permoda-Osip A, Dorszewska J, Bartkowska-Sniatkowska A, Chlopocka-Wozniak M, Rybakowski JK (2013). Vitamin B12 level may be related to the efficacy of single ketamine infusion in bipolar depression. *Pharmacopsychiatry*.

[105] Sun WL, Hua S, Li XY, Shen L, Wu H, Ji HF (2023). Microbially produced vitamin B12 contributes to the lipid-lowering effect of silymarin. *Nature Communications*.

[106] Brandt LJ, Bernstein LH, Wagle A (1977). Production of vitamin B 12 analogues in patients with small-bowel bacterial overgrowth. *Annals of Internal Medicine*.

[107] Finegold SM (1969). Intestinal bacteria. The role they play in normal physiology, pathologic physiology, and infection. *California Medicine*.

[108] Almeida OP, Ford AH, Hirani V, Singh V, vanBockxmeer FM, McCaul K (2014). B vitamins to enhance treatment response to antidepressants in middle-aged and older adults: results from the B-VITAGE randomised, double-blind, placebo-controlled trial. *The British Journal of Psychiatry*.

[109] Rakić M, Lunić T, Bekić M, Tomić S, Mitić K, Graovac S (2023). Vitamin B complex suppresses neuroinflammation in activated microglia: in vitro and in silico approach combined with dynamical modeling. *International Immunopharmacology*.

[110] Mandić M, Mitić K, Nedeljković P, Perić M, Božić B, Lunić T (2022). Vitamin B Complex and Experimental Autoimmune Encephalomyelitis -Attenuation of the Clinical Signs and Gut Microbiota Dysbiosis. *Nutrients*.

[111] Jolivalt CG, Mizisin LM, Nelson A, Cunha JM, Ramos KM, Bonke D (2009). B vitamins alleviate indices of neuropathic pain in diabetic rats. *European Journal of Pharmacology*.

[112] Gurwara S, Ajami NJ, Jang A, Hessel FC, Chen L, Plew S (2019). Dietary Nutrients Involved in One-Carbon Metabolism and Colonic Mucosa-Associated Gut Microbiome in Individuals with an Endoscopically Normal Colon. *Nutrients*.

